# Comparative efficacy and safety of acupoint injection-related therapies for the treatment of intractable hiccup after stroke: a network meta-analysis

**DOI:** 10.3389/fneur.2026.1698913

**Published:** 2026-02-10

**Authors:** Jun Tan, Ruihua Xu, Lixue Lin, Mengqing Luo, Jialei Ge, Jiabao Liu, Yan Ma, Wen Su, Rui Sun

**Affiliations:** 1 Department of Rehabilitation, Traditional Chinese and Western Medicine Hospital of Wuhan, Tongji Medical College, Huazhong University of Science and Technology, Wuhan, China; 2 College of Acupuncture-Moxibustion and Orthopedics, Hubei University of Chinese Medicine, Wuhan, China; 3 Wuhan Wuchang Hospital, Wuhan, China

**Keywords:** acupoint injection, CV12, intractable hiccups, ST36, stroke

## Abstract

**Objective:**

To systematically evaluate the effectiveness of various therapies (acupoint injection, acupuncture, etc.) in alleviating intractable hiccups after stroke through a comprehensive systematic review and network meta-analysis.

**Methods:**

We searched multiple databases up to July 2025 to find clinical randomized controlled trials on acupoint injection-related therapies for post-stroke intractable hiccups. Three authors independently screened studies. The quality of included studies was assessed according to PRISMA guidelines using the Cochrane RoB2 tool. Traditional meta-analyses of binary outcomes were performed with a fixed-effects model in Stata 17.0, complemented by prespecified subgroup analyses; comparative efficacy across interventions was subsequently estimated via network meta-analysis.

**Results:**

Twenty-one studies with 2,127 participants were included, and 17 studies with 1,658 participants were in the meta-analysis. The 95% confidence interval for the odds ratio of treatment effectiveness was (3.10, 7.63), indicating acupoint injection-related therapies were more effective than non-acupoint injection ones. The NMA of all 21 studies found that the combination of conventional acupuncture with auricular acupuncture exhibited the highest treatment efficacy (SUCRA 86.5%). This was closely followed by acupoint injection integrated with Western medical therapy (SUCRA 85.3%), while the regimen combining acupoint injection with standard acupuncture yielded a more modest efficacy profile (SUCRA 71.9%). However, the evidence quality of many interventions was low. Sensitivity analyses confirmed the findings’ robustness, and no publication bias was detected.

**Conclusion:**

Acupoint injection-related therapies, particularly the combined application of acupuncture, acupoint injection, auricular therapy, and Western medications, demonstrate significant efficacy in alleviating intractable hiccups after stroke. This approach offers valuable reference and guidance for clinical treatment.

**Systematic review registration:**

https://www.crd.york.ac.uk/PROSPERO, Identifier CRD42024601310.

## 1 Introduction

Stroke is the second leading cause of death globally and a major contributor to severe disability. With the increasing trend of an aging population, the incidence of stroke is rising. Data indicate that approximately 5.5 million people die from stroke each year (32, 33). According to 2019 statistics, ischemic stroke is the most common type, accounting for 62.4% of all stroke cases (1). Hiccups are frequently observed in stroke patients, particularly those with ischemic stroke. When hiccups persist for more than 48 h, they are classified as refractory hiccups, which can significantly impair the patient’s quality of life. Studies have shown that hiccups may sometimes be an early sign of stroke, especially in cases associated with lateral brainstem syndrome (34). Hiccups are closely linked to neurological dysfunction, primarily characterized by involuntary contraction of the diaphragm and inspiratory movements. It has been suggested that hiccups may be associated with abnormal functioning of the respiratory control regions of the brain, particularly the medulla oblongata (2, 3). In stroke patients, intracranial injury may lead to dysregulation of the hiccup reflex, triggering persistent hiccups. Intractable hiccups not only impair the patient’s physical health but also affect their psychological well-being. Persistent hiccups may interfere with eating and communication, thereby impacting socialization and overall psychological health. Intractable hiccups are likely more prevalent than previously thought and involve multiple medical domains.

In the realm of treatment, a variety of pharmacological agents have demonstrated some efficacy in managing symptoms, including dopamine-blocking drugs, baclofen, gabapentin, and anticonvulsants (4). However, the transient nature of these medications’ effectiveness, coupled with a high incidence of adverse effects, underscores the urgent need for more effective therapeutic options. Traditional approaches, such as the administration of traditional Chinese medicine, acupuncture, moxibustion, and acupoint injections, have been shown to alleviate symptoms of intractable hiccups after stroke and shorten the duration of treatment. Yet, high-quality evaluative evidence in this domain remains limited. By employing a network meta-analysis (NMA) approach, this study synthesizes both direct and indirect evidence from multiple studies to comprehensively assess the effectiveness of acupoint injection-related therapies in improving symptoms of intractable hiccups following stroke. This analysis aims to provide valuable insights and guidance for clinical management.

## 2 Methods

### 2.1 Protocol registration

The protocol was developed in accordance with the Preferred Reporting Items for Systematic Reviews and meta-analyses (PRISMA) guidelines. Furthermore, the protocol has been successfully registered on the PROSPERO platform (Registration Number: CRD42024601310).

### 2.2 Search strategy

We conducted a comprehensive search of journal articles published in PubMed, Embase, Cochrane Library, China Biomedical Literature Service (CBM), China National Knowledge Infrastructure (CNKI), Wipo Database (VIP), and Wanfang Database up to July 2025. The search utilized keywords such as “stroke,” “cerebral infarction,” “cerebral hemorrhage,” “intractable hiccups,” and “acupoint injection.” The final search strategy was developed by combining subject headings, dependent terms, and free-text terms. Additionally, the reference lists of all selected articles were independently reviewed to identify any relevant studies that may have been overlooked in the initial search (Supplementary document 1).

### 2.3 Research qualifications

We employed note express as a tool to organize the research information obtained. Two researchers, JT and RX, conducted a comprehensive literature review, extracted relevant information, and cross-validated the findings. Any discrepancies were resolved through in-depth discussions or consultations with a third researcher, LL. During the literature screening process, article titles were initially reviewed, and those deemed irrelevant were excluded. Subsequently, the abstracts and full texts of the remaining articles were thoroughly examined to determine their eligibility for inclusion. In cases where critical information was unclear, the authors of the original studies were contacted via email or telephone to obtain the necessary details. The data extracted from the included studies encompassed the following aspects: demographic characteristics of the study population, types of interventions and control groups, number of subjects in each group (intervention vs. control), duration of follow-up in randomized controlled trials, and outcome indicators. Data collection specifically included: (1) basic information such as authors and publication dates, (2) baseline characteristics and interventions, (3) fundamental risk-of-bias assessment indicators, and (4) outcome indicator measurement data.

## 3 Eligibility

### 3.1 Type of study

We restricted eligibility to publicly accessible randomized controlled trials whose individual treatment arms contained ≥30 participants and whose overall enrolment reached ≥60. There was no restriction regarding the use of blinding, and studies were limited to those published in Chinese or English. Eligible studies included single-arm or multi-arm RCTs, full-text journal publications, and all types of trials conducted in the healthcare field. The following types of studies were excluded: dissertations, conference proceedings, case reports, retrospective studies, cohort studies, reviews, systematic evaluations, and meta-analyses. Studies were also excluded if they had incomplete data, did not report relevant data, or if odds ratios (OR) with 95% confidence intervals (CI) for the indicators or specific incidence rates could not be calculated. In cases of duplicate publications, only the earliest publication was selected for inclusion, while subsequent duplicates were excluded. Publications deemed to be of lower quality were excluded outright.

### 3.2 Type of participants

Participants included in the study must be patients aged 18 years and above who have been diagnosed with stroke and are experiencing intractable hiccups.

### 3.3 Types of intervention

Interventions were based on the combination of traditional Chinese medicine (TCM) and Western medicine therapies, incorporating principles of TCM theory and meridian point theory. These interventions included acupoint injections, TCM compound formulations, acupuncture, and Western medications. There were no restrictions on the timing of intervention initiation, treatment duration, selection of Chinese herbs, or choice of acupoints.

### 3.4 Type of outcome measure

The primary outcome measure was clinical treatment effectiveness.

## 4 Quality of the study

Two researchers (JT and RX) independently assessed the risk of bias using the methodology specified in the Cochrane Handbook for Systematic Reviews of Interventions. Any inconsistencies were resolved through consultation with a third researcher (LL). We employed the Cochrane Risk of Bias 2 (RoB 2) tool, revised in 2019, to evaluate the risk of bias in the included studies. The RoB 2 tool assessed the studies across the following five domains: (1) bias arising from the randomization process, (2) bias due to deviations from intended interventions, (3) bias resulting from missing outcome data, (4) bias in measurement of the outcome, and (5) bias in selection of the reported result.

## 5 Statistical analysis

The outcome indicators in this study were all dichotomous variables. The odds ratio (OR) was used as the effect size, and its 95% confidence interval (CI) was calculated. Given the heterogeneity among studies, a random effects model was chosen for data analysis. Network meta-analysis (NMA) was performed using the Network Package command in Stata 17.0 software. The network of evidence graph displays the number of patients who received each intervention, with larger dots indicating a greater number of patients. The thickness of the connecting lines between interventions represents the number of studies included. The Surface under the Cumulative Ranking Area (SUCRA) is presented as a percentage (range 0%–100%) to illustrate the relative effectiveness of each intervention, with higher percentages indicating greater efficacy. Additionally, “comparison-corrected” funnel plots were used to assess publication bias and small-sample effects in the included studies.

## 6 Results

### 6.1 Literature search results

**Figure 1:**
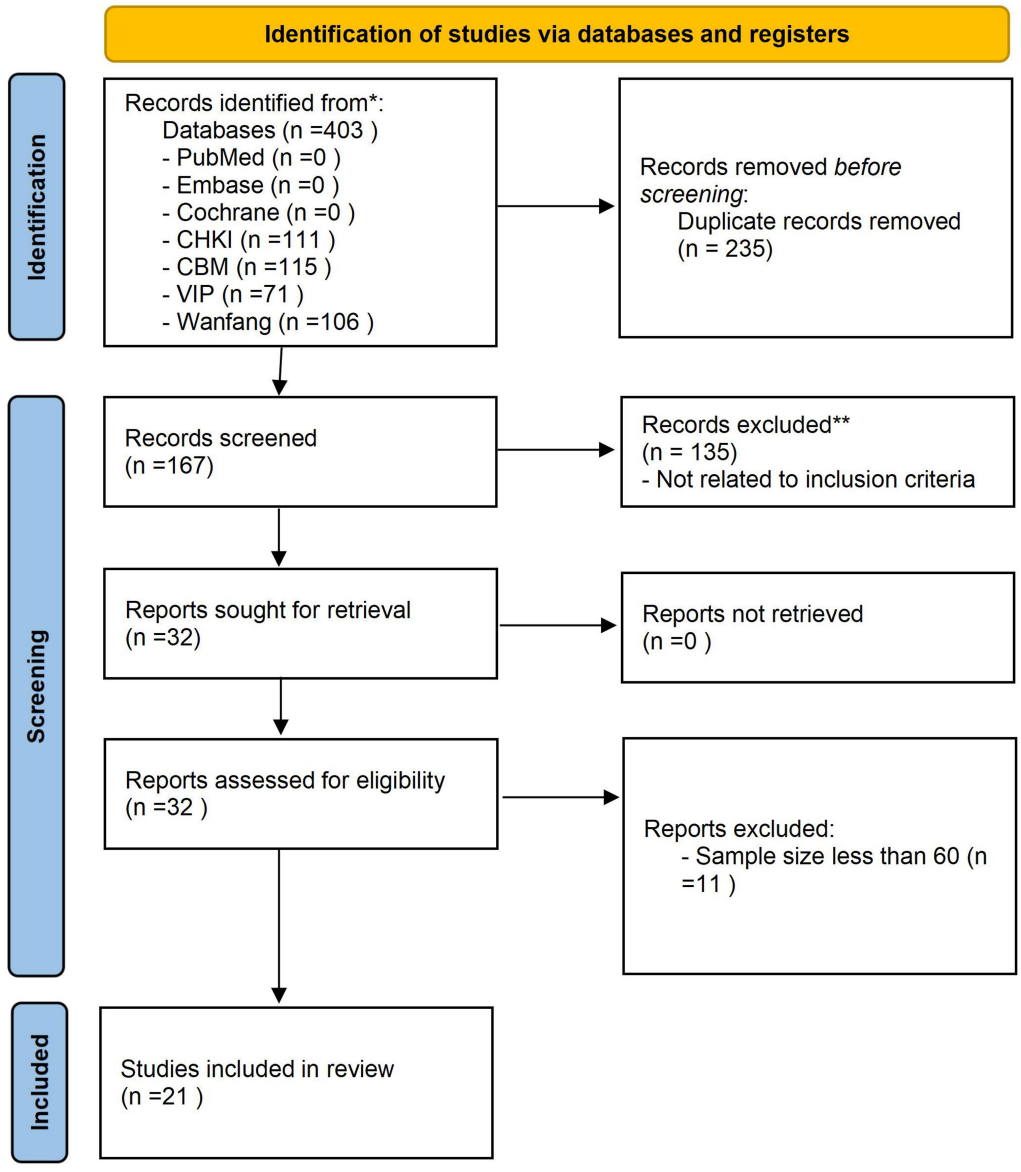
Flow chart of the study selection process.

Figure 1 presents the PRISMA flowchart for the systematic review. In the initial screening phase, a comprehensive search of the databases yielded a total of 403 relevant papers. Using NoteExpress software, we removed 235 duplicate citations, resulting in 167 unique title and abstract citations. Further screening led to the exclusion of 135 records: 12 conference abstracts, 14 theses, 22 reviews, 2 case reports, 3 off-topic papers, 49 non-randomized or single-arm trials, and 33 studies with ambiguous diagnostic or intervention descriptions. Of the 32 full-text articles that advanced to eligibility assessment, 11 were eliminated because their single-group sample sizes were <30. After additional screening, 21 studies were included in the analysis, with 17 studies incorporated into the meta-analysis and all 21 studies subjected to network meta-analysis (NMA).

### 6.2 Characteristics of included studies

A total of 21 randomized controlled trials (RCTs) published in Chinese were included in the analysis. All studies were two-arm trials except for one, which was a three-arm trial. The studies collectively involved 2,127 participants, with 1,118 assigned to the intervention groups and 1,009 to the control groups. The included studies were published between 2004 and 2024. Eleven different interventions were represented, including electroacupuncture, Western medicine (5–17), acupoint injections (5–17), acupoint injections combined with electroacupuncture (15, 16, 18), acupoint injections combined with hot moxibustion (6), acupoint injections combined with Western medicine (17), acupoint injections combined with acupuncture (17), acupoint injections combined with traditional Chinese medicine (5, 10, 14, 19), acupuncture alone (20, 21), acupuncture combined with auricular acupuncture (22), and Chinese medicine (23), The primary outcome measure for the included studies was treatment effectiveness. Detailed information on the included studies is presented in Table 1.

**Table 1:** Summary of the characteristics of the included studies.

Author (year)	Sample size	Age ( $\bar{x}$ ±s)	Gender (male/female)	Medicine	Acupuncture point	Intervention
Juke Wang (24)	86	61.1 ± 5.12	51/35	Phenobarbital	ST36	XZ+ZC/XZ
Ruobin et al. (10)	65	59.36 ± 4.93	36/29	Metoclopramide	ST36	XZ+ZY/XY
Honglei et al. (22)	98	61.16 ± 12.90	42/46	Metoclopramide	ST36	XZ/ZC+EX
Yuan et al. (6)	66	58.8 ± 8.30	45/21	Metoclopramide	ST36	XZ+JF/XY
Peng et al. (31)	98	58.8 ± 8.32	55/43	Metoclopramide	ST36	XZ+ZC/XZ
Ruchun et al. (7)	120	66.75 ± 10.98	47/73	Metoclopramide	ST36, PC6	XZ+ZC/XY
Xin et al. (8)	68	/	38/30	Raceanisodamine	ST36, BL17	XZ/XY
Peng Xiang et al. (9)	160	70.5 ± 3.6	88/72	Chlorpromazine	PC6	XZ/XY
Ying et al. (10)	70	62.15 ± 11.16	43/27	VB1, VB12	ST36	XZ+ZY/XY
Chaoqin et al. (23)	225	/	125/100	VB6	Ah Shi point	XZ/ZC/ZY
Shan et al. (19)	60	/	45/15	Metoclopramide	ST36	XZ+ZY/XZ
Jing et al. (11)	86	55.57 ± 11.96	47/39	VB1, VB2	BL17	XZ+ZC/XY
Shu et al. (12)	72	55.05 ± 12.0	57/15	Lidocaine	ST36, PC6	XZ/XY
Yamei et al. (13)	86	/	46/40	Chlorpromazine	PC6	XZ/XY
Lihua et al. (14)	60	/	46/14	Metoclopramide	ST36	XZ+ZY/XY
Chaoyang et al. (20)	62	/	40/22	Metoclopramide	ST36	XZ/ZC
Ying et al. (15)	160	/	100/60	VB1+VB2+Raceanisodamine	PC6, ST36	XZ+DZ/DZ
Ying et al. (18)	150	/	94/56	VB1, VB12	ST36	XZ+DZ/XY
Yifei et al. (16)	120	/	75/45	VB1, VB12	ST36, PC6	XZ+DZ/XY
Junmin et al. (17)	153	/	79/74	Metoclopramide	PC6, ST36, BL17, CV12, CV17, and CV22	XZ+XY/XY
Airong et al. (21)	62	/	42/20	Raceanisodamine	BL17	XZ/ZC

DZ, Electroacupuncture; XY, Western medicine; XZ, Acupoint injection; XZ+DZ, Acupoint injection and Electroacupuncture; XZ+JF, Acupoint injection and Hot moxibustion; XZ+XY, Acupoint injection and Western medicine; XZ+ZC, Acupoint injection and Acupuncture; XZ+ZY, Acupoint injection and Chinese medicine; ZC, Acupuncture; ZC+EX, Acupuncture and Auricular therapy; ZY, Chinese medicine.

### 6.3 Risk of bias of included studies

**Figure 2:**
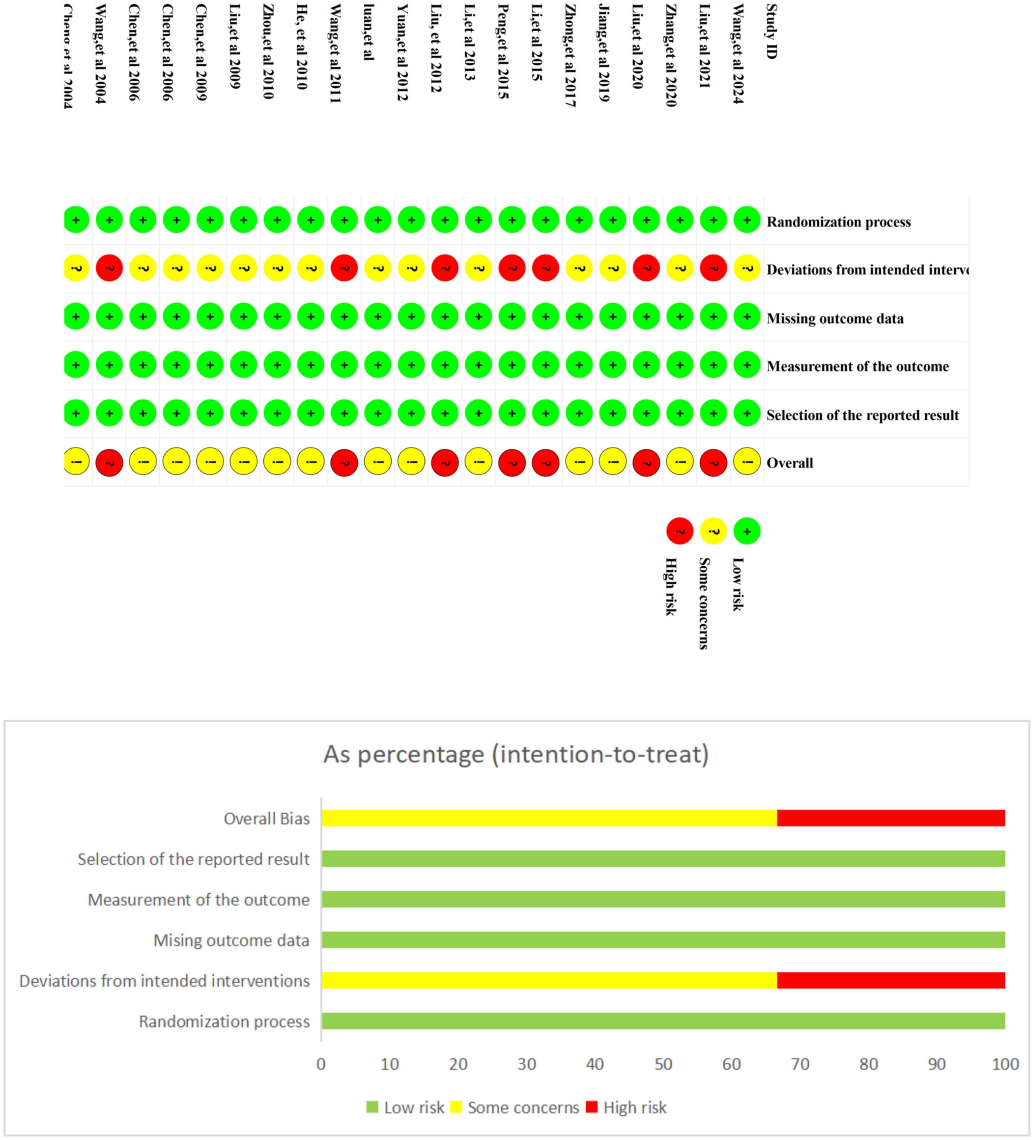
Risk of bias assessment.

The risk of bias in the included studies was assessed using the Cochrane Risk of Bias 2 (RoB 2) tool (Figure 2). Among the included studies, only two (7, 24) explicitly used a randomized table of numbers to generate the randomization sequence. The remaining 19 studies (7, 24) mentioned randomization but did not specify the method used. Additionally, none of the studies provided details regarding allocation concealment Table 2.

**Table 2:** The result of NMA for intractable hiccups after stroke.

_ref_	__y_ZY_	__y_ZC+EX_	__y_ZC_	__y_XZ+ZY_	__y_XZ+ZC_	__y_XZ+XY_	__y_XZ+JF_	__y_XZ+DZ_	__y_XZ_	__y_XY_
ref	−0.88 (−3.09, 1.34)	2.68 (0.08, 5.28)	−0.44 (−2.62, 1.74)	1.49 (−0.65, 3.64)	1.96 (−0.13, 4.04)	2.56 (0.22, 4.89)	2.04 (−0.89, 4.97)	1.93 (0.40, 3.46)	1.02 (−1.04, 3.08)	−0.11 (−2.09, 1.87)
0.88 (−1.34, 3.09)	_y_ZY	3.56 (1.78, 5.34)	0.43 (−0.23, 1.10)	2.37 (1.11, 3.62)	2.83 (1.76, 3.90)	3.43 (1.84, 5.02)	2.92 (0.54, 5.29)	2.80 (1.21, 4.40)	1.89 (1.09, 2.70)	0.76 (−0.23, 1.75)
−2.68 (−5.28, −0.08)	−3.56 (−5.34, −1.78)	_y_ZC+EX	−3.12 (−4.86, −1.38)	−1.19 (−3.05, 0.67)	−0.72 (−2.46, 1.02)	−0.13 (−2.23, 1.97)	−0.64 (−3.38, 2.10)	−0.75 (−2.86, 1.35)	−1.67 (−3.25, −0.08)	−2.79 (−4.48, −1.10)
0.44 (−1.74, 2.62)	−0.43 (−1.10, 0.23)	3.12 (1.38, 4.86)	_y_ZC	1.93 (0.74, 3.13)	2.40 (1.40, 3.40)	3.00 (1.45, 4.54)	2.48 (0.14, 4.83)	2.37 (0.82, 3.92)	1.46 (0.75, 2.17)	0.33 (−0.59, 1.24)
−1.49 (−3.64, 0.65)	−2.37 (−3.62, −1.11)	1.19 (−0.67, 3.05)	−1.93 (−3.13, −0.74)	_y_XZ+ZY	0.47 (−0.57, 1.51)	1.06 (−0.43, 2.56)	0.55 (−1.76, 2.86)	0.44 (−1.06, 1.94)	−0.47 (−1.43, 0.48)	−1.60 (−2.43, −0.78)
−1.96 (−4.04, 0.13)	−2.83 (−3.90, −1.76)	0.72 (−1.02, 2.46)	−2.40 (−3.40, −1.40)	−0.47 (−1.51, 0.57)	_y_XZ+ZC	0.60 (−0.81, 2.01)	0.08 (−2.17, 2.34)	−0.03 (−1.44, 1.39)	−0.94 (−1.65, −0.23)	−2.07 (−2.73, −1.41)
−2.56 (−4.89,−0.22)	−3.43 (−5.02,−1.84)	0.13 (−1.97, 2.23)	−3.00 (−4.54, −1.45)	−1.06 (−2.56, 0.43)	−0.60 (−2.01, 0.81)	_y_XZ+XY	−0.51 (−3.00, 1.98)	−0.63 (−2.39, 1.14)	−1.54 (−2.91, −0.17)	−2.67 (−3.91, −1.42)
−2.04 (−4.97, 0.89)	−2.92 (−5.29, −0.54)	0.64 (−2.10, 3.38)	−2.48 (−4.83, −0.14)	−0.55 (−2.86, 1.76)	−0.08 (−2.34, 2.17)	0.51 (−1.98, 3.00)	_y_XZ+JF	−0.11 (−2.61, 2.38)	−1.03 (−3.26, 1.21)	−2.15 (−4.31, 0.00)
−1.93 (−3.46, −0.40)	−2.80 (−4.40, −1.21)	0.75 (−1.35, 2.86)	−2.37 (−3.92, −0.82)	−0.44 (−1.94, 1.06)	0.03 (−1.39, 1.44)	0.63 (−1.14, 2.39)	0.11 (−2.38, 2.61)	_y_XZ+DZ	−0.91 (−2.29, 0.47)	−2.04 (−3.29, −0.79)
−1.02 (−3.08, 1.04)	−1.89 (−2.70, −1.09)	1.67 (0.08, 3.25)	−1.46 (−2.17, −0.75)	0.47 (−0.48, 1.43)	0.94 (0.23, 1.65)	1.54 (0.17, 2.91)	1.03 (−1.21, 3.26)	0.91 (−0.47, 2.29)	_y_XZ	−1.13 (−1.71, −0.55)
0.11 (−1.87, 2.09)	−0.76 (−1.75, 0.23)	2.79 (1.10, 4.48)	−0.33 (−1.24, 0.59)	1.60 (0.78, 2.43)	2.07 (1.41, 2.73)	2.67 (1.42, 3.91)	2.15 (−0.00, 4.31)	2.04 (0.79, 3.29)	1.13 (0.55, 1.71)	_y_XY

### 6.4 Meta-analysis

**Figure 3:**
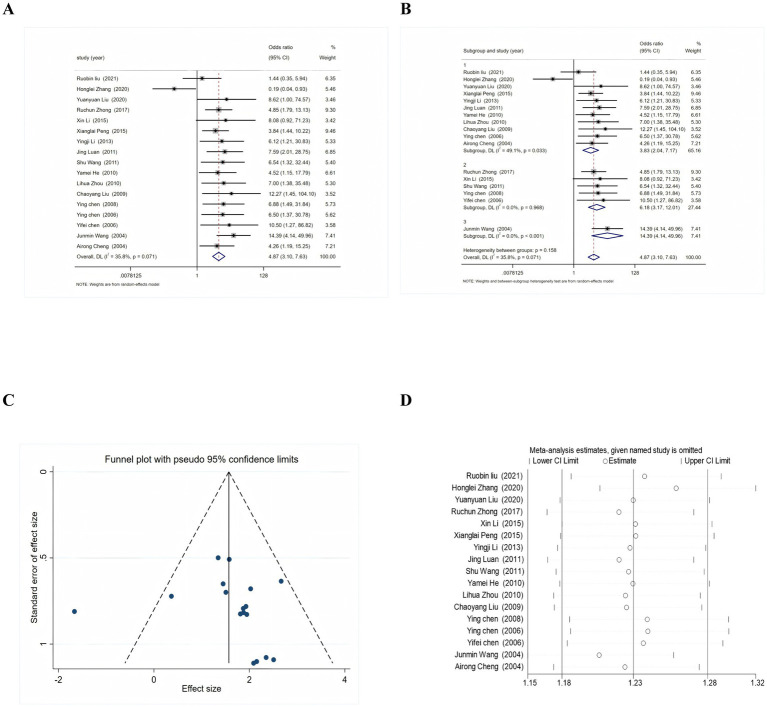
Results of meta-analysis of the effectiveness of acupoint injection therapy on recalcitrant hiccups after stroke. **(A)** Forest plot of the effect of acupoint injection therapy on recalcitrant hiccups after stroke. **(B)** Results of subgroup analysis of the number of acupoints on the efficacy of acupoint injection therapy. **(C)** Results of funnel plot analysis of acupoint injection therapy. **(D)** Results of sensitivity analysis of acupoint injection therapy.

Seventeen studies, involving a total of 1,658 participants, were included to assess the impact of acupoint injection therapy on the efficacy of intractable hiccups after stroke (Figure 3A). The meta-analysis revealed that the efficacy level was significantly higher in the acupoint-related therapy group, with an odds ratio (OR) of 4.87 [95% confidence interval (CI): 3.10, 7.63]. Inter-study heterogeneity was minimal and non-significant (*I*^2^ = 35.8%, *p* = 0.071) (Figure 3B). Subgroup analyses of studies with 1 injection point, 2 injection points, and more than 2 injection points also demonstrated low heterogeneity (*I*^2^ < 50%). The funnel plot suggested the presence of publication bias (Figure 3C). However, sensitivity analyses conducted by sequentially excluding each study one by one indicated that the combined results of the remaining studies remained statistically significant (95% CI excluding 1). This finding suggests that the results of the original meta-analysis were robust and not significantly influenced by the inclusion or exclusion of individual studies (Figure 3D). Potential causes of publication bias may include low-quality small-sample experiments, true heterogeneity, artifacts, and chance.

### 6.5 Network meta-analysis

**Figure 4:**
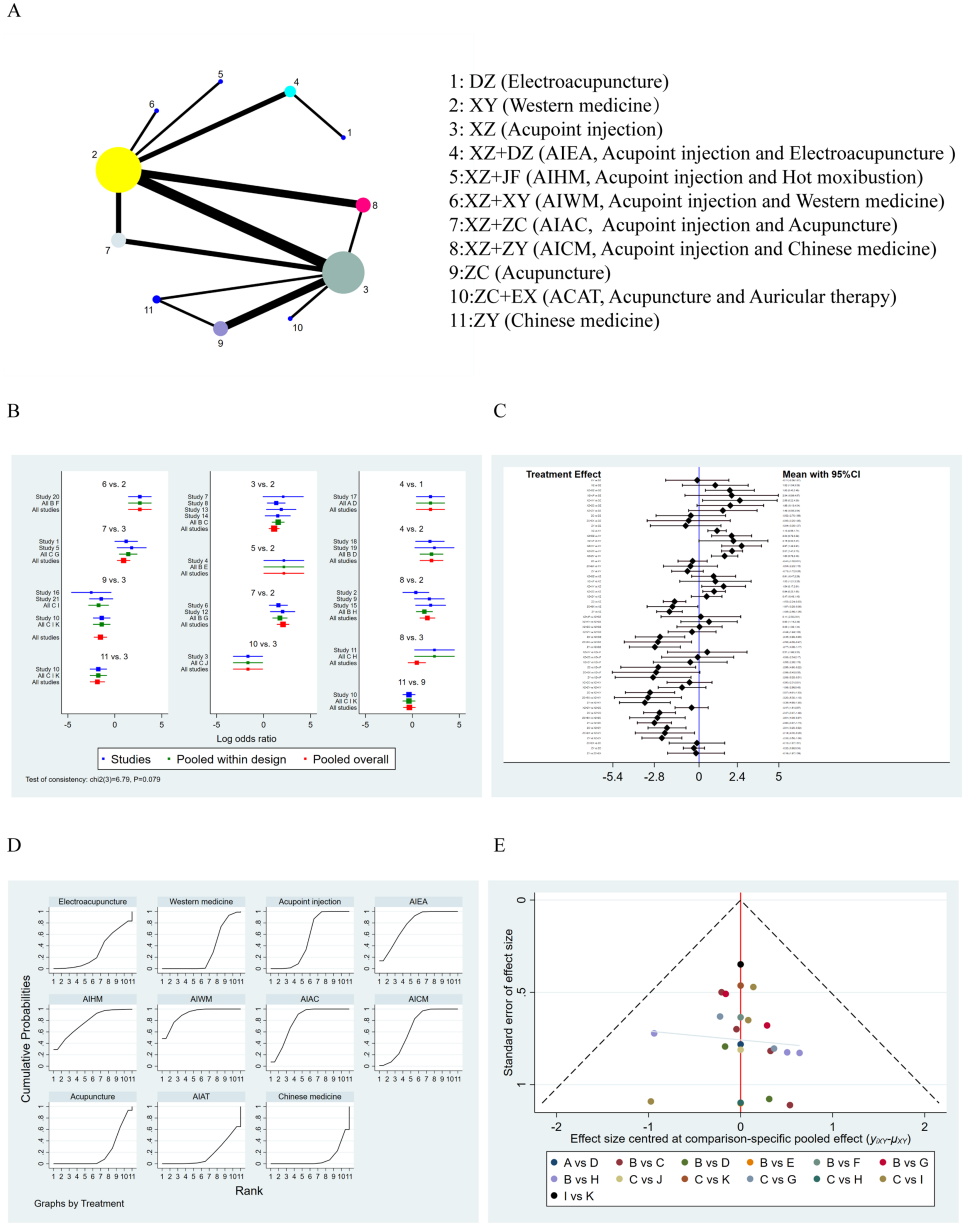
Results of a reticulation meta-analysis of the effectiveness of acupoint injection-related therapies for intractable hiccups after stroke. **(A)** Network plot of 11 treatment options; **(B)** Forest plot; **(C)** Two-by-two comparison forest plot; **(D)** Cumulative probability plot; **(E)** Funnel plot.

A total of 21 studies involving 2,127 participants assessed the effects of acupoint injection-related therapies on treatment efficacy. The network meta-analysis revealed that Western medicine, acupoint injection, acupoint injection + acupuncture, and acupoint injection + traditional Chinese medicine formed a closed loop with both direct and indirect comparisons (Figure 4A). The forest plot (Figure 4B) showed that the inconsistency test results were not statistically significant (*p* > 0.05). Both the loop test and the node-splitting method suggested fair consistency (*p* > 0.05).

The results of pairwise comparisons (Figure 4C) indicated the following: (1) The efficacy of acupoint injections combined with electroacupuncture (OR = 1.93; 95% CI: 0.40, 3.48) and acupoint injections combined with Western medicine (OR = 2.58; 95% CI: 0.22, 4.89) was higher than that of electroacupuncture alone. (2) The efficacy of acupoint injection alone (OR = 1.13; 95% CI: 0.55, 1.71), acupoint injections combined with electroacupuncture (OR = 2.04; 95% CI: 0.79, 3.29), acupoint injections combined with Western medicine (OR = 2.67; 95% CI: 1.42, 3.91), acupoint injections combined with acupuncture (OR = 2.07; 95% CI: 1.41, 2.73), and acupoint injection combined with Chinese medicine (OR = 1.60; 95% CI: 0.78, 2.43) was higher than that of Western medicine alone. (3) The efficacy of acupoint injection combined with Western medicine (OR = 1.51; 95% CI: 0.17, 2.91) and acupoint injection combined with acupuncture (OR = 0.94; 95% CI: 0.23, 1.65) was higher than that of acupoint injection alone. In contrast, acupuncture alone (OR = −1.53; 95% CI: −2.24, −0.83), acupuncture combined with auricular acupuncture (OR = −16.7; 95% CI: −3.25, −0.08), and Chinese medicine alone (OR = −1.85; 95% CI: −2.66, −1.05) were less efficacious than acupoint injection alone. (4) Acupuncture alone (OR = −2.45; 95% CI: −3.99, −0.90), acupuncture combined with auricular acupuncture (OR = −2.58; 95% CI: −4.68, −0.47), and Chinese medicine alone (OR = −2.77; 95% CI: −4.36, −1.17) were less efficacious than acupoint injection combined with electroacupuncture.(5) Acupuncture alone (OR = −2.56; 95% CI: −4.90, −0.22) and Chinese medicine alone (OR = −2.88; 95% CI: −5.25, −0.51) were less efficacious than acupoint injection combined with hot moxibustion.(6) Acupuncture alone (OR = −3.07; 95% CI: −4.61, −1.53), acupuncture combined with hot auricular acupuncture (OR = −3.20; 95% CI: −5.30, −1.10), and Chinese medicine alone (OR = −3.39; 95% CI: −4.98, −1.80) were less efficacious than acupoint injection + Western medicine.(7) Acupuncture alone (OR = −2.47; 95% CI: −3.47, −1.48), acupuncture combined with auricular acupuncture (OR = −2.61; 95% CI: −4.35, −0.87), and Chinese medicine alone (OR = −2.80; 95% CI: −3.87, −1.73) were less efficacious than acupoint injection combined with acupuncture.(8) Acupuncture alone (OR = −2.01; 95% CI: −3.20, −0.82), acupuncture combined with auricular acupuncture (OR = −2.14; 95% CI: −4.00, −0.28), and Chinese medicine alone (OR = −2.33; 95% CI: −3.58, −1.08) were less efficacious than acupoint injection combined with Chinese medicine.

According to the SUCRA results (Figure 4D), the ranking of interventions based on efficacy was as follows: acupuncture + auricular acupuncture (SUCRA: 86.5%), acupoint injection + Western medicine (SUCRA: 85.3%), acupoint injection + acupuncture (SUCRA: 71.9%), acupoint injection + moxibustion (SUCRA: 71.1%), acupoint injection + electroacupuncture (SUCRA: 70.2%), acupoint injection + Chinese medicine (SUCRA: 57.1%), acupoint injection alone (SUCRA: 43.4%), electroacupuncture alone (SUCRA: 23.7%), Western medicine alone (SUCRA: 21.8%), acupuncture alone (SUCRA: 15.2%), and traditional Chinese medicine alone (SUCRA: 3.9%). The funnel plot results (Figure 4E) showed no evidence of publication bias in the efficacy outcome analysis across the 21 studies.

## 7 Discussion

### 7.1 Summary of findings

This network meta-analysis (NMA) evaluated the effectiveness of acupoint injection-related therapies in improving persistent intractable hiccups. A total of 21 studies involving 2,127 participants were included. The results demonstrated that acupoint injection-related therapies significantly enhanced treatment efficacy in patients with intractable hiccups. Specifically, the NMA revealed that the combination of acupuncture and auricular acupuncture was the most effective intervention for alleviating intractable hiccups after stroke, followed by acupoint injection combined with Western drug therapy, and then acupoint injection combined with acupuncture. Heterogeneity within this network meta-analysis was low, and subgroup analyses did not identify a significant effect of the number of acupoints on intervention outcomes. Based on the available evidence, acupuncture combined with auricular acupoint therapy is recommended as the preferred strategy for improving in intractable hiccups, with acupoint injection combined with Western medicine as the second-best option.

To facilitate the clinical application of acupoint injection-related therapies, we summarized the specific methods of the various interventions covered in the literature, including the acupoints used and the medications administered. The most frequently utilized acupoints were from the Foot Yangming Stomach Meridian (ST36, used in 16 studies) and the Hand Jueyin Pericardium Meridian (PC6, used in 7 studies). ST36 is a key point on the Foot Yangming Stomach Meridian, serving as both the lower conjunction point of the stomach among the six viscera and the conjunction point of the Stomach Meridian. As documented in “Lingshu—Evil Qi and Viscera Disease Formation,” “Xingtong governs the external meridians and He governs the internal organs.” Additionally, “Difficulties—Sixty-eight Difficulties” states that “He governs rebellious qi and leakage,” highlighting its unique therapeutic effect on intractable hiccups caused by the upward reversal of stomach qi. PC6, a complex point on the Hand Jueyin Pericardium Meridian, originates from the middle of the chest, descends through the diaphragm, and passes through the triple burner.

For acupoint injections, the most commonly used drug was Metoclopramide, followed by a combination of Vitamin B1 and Vitamin B12. Other studies have suggested that baclofen and gabapentin can be considered first-line treatments for persistent and intractable hiccups, with metoclopramide and chlorpromazine as alternative options (25).

### 7.2 Quality of evidence

Our assessment revealed that each of the included studies exhibited a high risk of bias, potentially leading to false-positive results. Blinding is a crucial factor in determining the risk of bias in a study; however, most studies failed to provide detailed information regarding blinding procedures. Furthermore, all studies were conducted in China, which limits the generalizability of the findings. Therefore, additional independent studies conducted in diverse countries are essential to validate the broader applicability of these results.

### 7.3 Limitations

Several limitations are acknowledged in this review. First, there is a notable lack of data on critical clinical outcomes, such as the frequency and duration of intractable hiccups episodes. This deficiency significantly impedes the ability to conduct a comprehensive pooled analysis of the results. Additionally, most studies utilized response rate as the primary outcome metric, which may vary due to differences in operational practices among practitioners. Future studies should therefore consider selecting more robust and standardized outcome indicators. Second, the quality of the included randomized controlled trials (RCTs) was inconsistent, which may compromise the reliability of the positive findings. Many of the included studies were rated as having “some concerns” in the risk-of-bias assessment, indicating that caution is warranted when interpreting the results of this study. This study was conducted in China and published in Chinese, presenting regional bias that limits its global applicability. The findings primarily reflect Chinese patients’ responses to acupuncture, and caution is advised when extrapolating results to other populations. The intervention measures and evaluation criteria are based on China’s healthcare system and may not be applicable to international practice. Furthermore, the majority of the included studies did not assess the incidence of adverse events. Future studies involving acupuncture and acupoint injection therapies should prioritize investigating the incidence of adverse events and exploring their potential associations with these interventions.

### 7.4 Implications for future research

Future research on acupoint injection-related therapies for the treatment of persistent intractable hiccups should prioritize the conduct of randomized controlled trials (RCTs) employing rigorous methodologies. Pilot trials should be utilized to optimize the design of these RCTs. The trial design, execution, and reporting must adhere strictly to the Consolidated Standards for Reporting Trials (CONSORT) and STRICTA (Standards for Reporting Interventions in Clinical Trials of Acupuncture) guidelines. In addition to these standards, factors that may influence clinical heterogeneity should be carefully considered during the trial design phase. These factors include acupoint selection, treatment duration, intervention duration, treatment frequency, and intervention dose. Long-term studies are essential to assess the durability of treatment effects. We recommend longitudinal trials to thoroughly investigate the long-term efficacy and safety of acupoint injections for the treatment of intractable hiccups after stroke. Concurrently, we suggest future multinational, multicenter collaborative studies to validate the cross-regional applicability of these treatment outcomes, thereby enhancing the study’s universal value.

## 8 Summary

This review highlights that, among acupoint injection-related therapies, the combination of acupuncture with auricular acupuncture demonstrated the highest SUCRA probability (86.5%) for treating intractable hiccups after stroke. This was followed by acupoint injection combined with Western medicine (SUCRA: 85.3%), while acupoint injection combined with acupuncture ranked third (SUCRA: 71.9%). Stroke patients often present with multiple complications, including gastrointestinal dysfunction, hemiplegia, anxiety, and insomnia. The integration of acupuncture and auricular therapy not only alleviates hiccups but also exerts broader therapeutic effects. Conventional acupuncture modulates gastrointestinal motility by stimulating specific acupoints such as Zusanli (ST36) and Zhongwan (CV12), while auricular therapy—*via* points such as Shenmen (TE1) and Sympathetic—promotes sedation and neural regulation. This synergistic approach contributes to comprehensive improvements in patients’ quality of life.

However, treatment efficacy is influenced by factors such as stroke location, disease duration (particularly the duration of hiccups), and individual syndrome patterns. In some cases, patients with severe neural damage or physical debilitation may exhibit a diminished response to body acupuncture and limited efficacy of auricular therapy, resulting in suboptimal outcomes. Additionally, prolonged stimulation of auricular points may lead to local skin irritation, erythema, swelling, or pain; excessive needling depth may damage auricular cartilage and potentially induce localized inflammation.

The pharmacological agents used in acupoint injections are largely consistent with Western medical standards, with metoclopramide being the most commonly employed. While these medications may offer short-term symptomatic relief, they are associated with a relatively high incidence of adverse effects. By integrating pharmacological intervention with the regulatory mechanisms of acupoints and meridians, this dual-modality approach targets both the symptoms and underlying pathophysiology of hiccups, thereby reducing recurrence rates and enhancing overall therapeutic outcomes.

This conclusion mirrors the report by Qian Mo et al., who showed that auricular acupuncture is the dominant micro-system technique and the most frequently applied modality. Among combination therapies, filiform needle plus acupoint injection is used most often (26). The neurobiology of persistent hiccups is complex, engaging multiple central and peripheral transmitters and circuits (4). Centrally, GABA, dopamine, and serotonin are the principal mediators; peripherally, epinephrine, norepinephrine, acetylcholine, and histamine are involved (27). The only drug currently licensed by the FDA for intractable hiccups is the dopamine D2-receptor antagonist chlorpromazine (35). Electroacupuncture at ST36 raises both local and cerebrospinal-fluid glutamate levels in rats, whereas PC6 stimulation increases tissue dopamine (28). Because glutamate is the immediate precursor of the ubiquitous inhibitory transmitter GABA, these observations provide a plausible neurochemical basis for acupuncture’s anti-hiccup effect.

The literature suggests that acupuncture, when used as an adjunct to pharmacological therapy, may significantly benefit patients with post-stroke hiccups (29, 30). However, the level of evidence is compromised by a high risk of bias, resulting in low or very low certainty in the findings. Thus, further well-designed studies are needed to validate the efficacy of acupoint injection combined with acupuncture for treating intractable hiccups after stroke.

## Data Availability

The original contributions presented in the study are included in the article/Supplementary material, further inquiries can be directed to the corresponding author.
