# Supplementary material for: Comparative efficacy and safety of acupoint injection-related therapies for the treatment of intractable hiccup after stroke: a network meta-analysis
Source: Front Neurol. 2026 Feb 10;17:1698913. doi: 10.3389/fneur.2026.1698913 (PMC12929137; doi:10.3389/fneur.2026.1698913)
Supplement: Supplementary file 1 [file Table_1.docx]

DATABASE: PubMed

RETRIEVE DATE: Build - July 9, 2025

RETRIEVAL STRATEGY:

Search: ****(((((Stroke) OR ((((((((((((((((((((((((((((Strokes[Title/Abstract]) OR (Cerebrovascular Accident[Title/Abstract])) OR (Cerebrovascular Accidents[Title/Abstract])) OR (Cerebral Stroke[Title/Abstract])) OR (Cerebral Strokes[Title/Abstract])) OR (Stroke, Cerebral[Title/Abstract])) OR (Strokes, Cerebral[Title/Abstract])) OR (Cerebrovascular Apoplexy[Title/Abstract])) OR (Apoplexy, Cerebrovascular[Title/Abstract])) OR (Vascular Accident, Brain[Title/Abstract])) OR (Brain Vascular Accident[Title/Abstract])) OR (Brain Vascular Accidents[Title/Abstract])) OR (Vascular Accidents, Brain[Title/Abstract])) OR (Cerebrovascular Stroke[Title/Abstract])) OR (Cerebrovascular Strokes[Title/Abstract])) OR (Stroke, Cerebrovascular[Title/Abstract])) OR (Strokes, Cerebrovascular[Title/Abstract])) OR (Apoplexy[Title/Abstract])) OR (CVA (Cerebrovascular Accident[Title/Abstract]))) OR (CVAs (Cerebrovascular Accident[Title/Abstract]))) OR (Stroke, Acute[Title/Abstract])) OR (Acute Stroke[Title/Abstract])) OR (Acute Strokes[Title/Abstract])) OR (Strokes, Acute[Title/Abstract])) OR (Cerebrovascular Accident, Acute[Title/Abstract])) OR (Acute Cerebrovascular Accident[Title/Abstract])) OR (Acute Cerebrovascular Accidents[Title/Abstract])) OR (Cerebrovascular Accidents, Acute[Title/Abstract]))) AND ((((Hiccups[Title/Abstract]) OR (Hiccough[Title/Abstract])) OR (Hiccoughs[Title/Abstract])) OR ("Hiccup"[Mesh]))) AND (("Acupuncture Points"[Mesh]) OR (((((Acupuncture Point[Title/Abstract]) OR (Point, Acupuncture[Title/Abstract])) OR (Points, Acupuncture[Title/Abstract])) OR (Acupoints[Title/Abstract])) OR (Acupoint[Title/Abstract])))) AND (("Injections"[Mesh]) OR (((Injection[Title/Abstract]) OR (Injectables[Title/Abstract])) OR (Injectable[Title/Abstract])))) AND ("Randomized Controlled Trial" [Publication Type])****

Database:Embase

Retrieval Date:Build - July 9, 2025

Retrieval Strategy:Embase

Session Results

.......................................................

No. Query Results Results Date

#17. #13 AND #16 9 Jul 2025

#16. #14 OR #15 1,127,129 9 Jul 2025

#15. 'controlled trial, randomized':ab,ti OR 228,275 9 Jul 2025

'randomised controlled study':ab,ti OR

'randomised controlled tria':ab,ti OR 'randomized

controlled study':ab,ti OR 'trial, randomized

controlled':ab,ti OR 'randomized controlled

trial':ab,ti

#14. 'randomized controlled trial'/exp 1,091,120 9 Jul 2025

#13. #9 AND #10 AND #11 AND #12 2 9 Jul 2025

#12. #7 OR #8 998,330 9 Jul 2025

#11. #5 OR #6 16,753 9 Jul 2025

#10. #3 OR #4 5,448 9 Jul 2025

#9. #1 OR #2 722,067 9 Jul 2025

#8. 'blood vessel injection':ab,ti OR 'gluteal 963,729 9 Jul 2025

injection':ab,ti OR 'injection solution':ab,ti OR

injections:ab,ti OR 'percutaneous

injection':ab,ti OR injection:ab,ti

#7. 'injection'/exp 185,108 9 Jul 2025

#6. 'acu point':ab,ti OR acupoint:ab,ti OR 13,449 9 Jul 2025

acupoints:ab,ti OR 'acupuncture points':ab,ti OR

'point, acupuncture':ab,ti OR 'acupuncture

point':ab,ti

#5. 'acupuncture point'/exp 8,369 9 Jul 2025

#4. hiccough:ab,ti OR singultus:ab,ti OR hiccup:ab,ti 1,211 9 Jul 2025

#3. 'hiccup'/exp 5,274 9 Jul 2025

#2. 'accident, cerebrovascular':ab,ti OR 'acute 588,120 9 Jul 2025

cerebrovascular lesion':ab,ti OR 'acute focal

cerebral vasculopathy':ab,ti OR 'acute

stroke':ab,ti OR 'apoplectic stroke':ab,ti OR

apoplexia:ab,ti OR apoplexy:ab,ti OR 'blood flow

disturbance, brain':ab,ti OR 'brain

accident':ab,ti OR 'brain attack':ab,ti OR 'brain

blood flow disturbance':ab,ti OR 'brain

insult':ab,ti OR 'brain insultus':ab,ti OR 'brain

vascular accident':ab,ti OR 'cerebral

apoplexia':ab,ti OR 'cerebral insult':ab,ti OR

'cerebral stroke':ab,ti OR 'cerebral vascular

accident':ab,ti OR 'cerebral vascular

insufficiency':ab,ti OR 'cerebro vascular

accident':ab,ti OR 'cerebrovascular arrest':ab,ti

OR 'cerebrovascular failure':ab,ti OR

'cerebrovascular injury':ab,ti OR

'cerebrovascular insufficiency':ab,ti OR

'cerebrovascular insult':ab,ti OR 'cerebrum

vascular accident':ab,ti OR 'cryptogenic

stroke':ab,ti OR cva:ab,ti OR 'insultus

cerebralis':ab,ti OR 'ischaemic seizure':ab,ti OR

'ischemic seizure':ab,ti OR stroke:ab,ti OR

'thrombotic stroke':ab,ti OR 'cerebrovascular

accident':ab,ti

#1. 'cerebrovascular accident'/exp 513,330 9 Jul 2025

.......................................................

DATABASE: Cochrane Library

SEARCH DATE: Build - July 9, 2025

SEARCH STRATEGY:

Search Name: Cochrane Library

Date Run: 09/07/2025 16:23:44

Comment:

ID Search Hits

#1 MeSH descriptor: [Stroke] explode all trees 18208

#2 (Stroke):ti,ab,kw OR (Strokes):ti,ab,kw OR (CVA (Cerebrovascular Accident)):ti,ab,kw OR (Apoplexy):ti,ab,kw OR (Cerebrovascular Accident):ti,ab,kw 80971

#3 (Stroke):ti,ab,kw OR (Cerebrovascular Stroke):ti,ab,kw OR (Vascular Accident, Brain):ti,ab,kw OR (Brain Vascular Accidents):ti,ab,kw OR (Cerebrovascular Strokes):ti,ab,kw 77226

#4 (Stroke):ti,ab,kw OR (Stroke, Cerebrovascular):ti,ab,kw OR (Cerebral Strokes):ti,ab,kw OR (Vascular Accidents, Brain):ti,ab,kw OR (Strokes, Cerebral):ti,ab,kw 77029

#5 (Stroke):ti,ab,kw OR (Cerebrovascular Accidents):ti,ab,kw OR (Strokes, Cerebrovascular):ti,ab,kw OR (Brain Vascular Accident):ti,ab,kw OR (Cerebral Stroke):ti,ab,kw 77433

#6 (Stroke):ti,ab,kw OR (Cerebrovascular Apoplexy):ti,ab,kw OR (Stroke, Cerebral):ti,ab,kw OR (Apoplexy, Cerebrovascular):ti,ab,kw OR (CVAs (Cerebrovascular Accident)):ti,ab,kw 76948

#7 (Stroke):ti,ab,kw OR (Cerebrovascular Accidents, Acute):ti,ab,kw OR (Cerebrovascular Accident, Acute):ti,ab,kw OR (Acute Strokes):ti,ab,kw OR (Acute Stroke):ti,ab,kw 77756

#8 (Stroke):ti,ab,kw OR (Acute Cerebrovascular Accidents):ti,ab,kw OR (Acute Cerebrovascular Accident):ti,ab,kw OR (Stroke, Acute):ti,ab,kw OR (Strokes, Acute):ti,ab,kw 77756

#9 #1 OR #2 OR #3 OR #4 OR #5 OR #6 OR #7 OR #8 81682

#10 MeSH descriptor: [Hiccup] explode all trees 28

#11 (Hiccup):ti,ab,kw OR (Hiccoughs):ti,ab,kw OR (Hiccough):ti,ab,kw OR (Hiccups):ti,ab,kw 473

#12 #10 OR #11 473

#13 MeSH descriptor: [Acupuncture Points] explode all trees 2860

#14 (Acupuncture Points):ti,ab,kw OR (Acupoint):ti,ab,kw OR (Point, Acupuncture):ti,ab,kw OR (Acupuncture Point):ti,ab,kw OR (Points, Acupuncture):ti,ab,kw 11187

#15 (Acupuncture Points):ti,ab,kw OR (Acupoints):ti,ab,kw 8637

#16 #13 OR #14 OR #15 12308

#17 MeSH descriptor: [Injections] explode all trees 27754

#18 (Injections):ti,ab,kw OR (Injectables):ti,ab,kw OR (Injectable):ti,ab,kw OR (Injection):ti,ab,kw 127138

#19 #17 OR #18 127208

#20 MeSH descriptor: [Randomized Controlled Trial] explode all trees 34

#21 #9 AND #12 AND #16 AND #19 AND #20 0

RETRIEVAL VIBRARY: CBM

RETRIEVAL DATE: Built - July 13, 2025

RETRIEVAL TYPE: (‘Acupuncture point injection’ [common field:smart] OR ‘Acupuncture point injection’ [common field:smart]) AND (‘Erratic’ [common field:smart]) AND (‘Stroke’ [common field:smart] OR ‘Stroke’ [common field:smart])

RETRIEVAL VALUE: CNKI

RETRIEVAL DATE: Build - July 13, 2025

RETRIEVAL TYPE: (SU = 'stroke' OR SU = 'stroke' ) AND (SU = 'hiccup' OR SU = 'hiccup' OR SU = 'hiccups' OR SU = 'intractable hiccups') AND (SU = 'Acupuncture injection' OR SU = 'acupoint injection therapy')

Retrieved from: VIP

Retrieved on: build date - July 13, 2025

Retrieved expression M = (stroke OR stroke) AND M = (hiccup OR hiccup OR hiccups) AND M = (acupoint injections OR acupoint injection therapy)

Retrieved from: Wanfang

Retrieved on: build date - July 13, 2025

Retrieved expression (subject line extension): title or keyword: (stroke OR Stroke) and Title or keyword:(eruption OR hiccup OR hiccup) and Title or keyword:(acupoint injection OR acupoint injection therapy)
